# Research on Stereolithography-Based Shape Memory Polymers/Fe_3_O_4_ Composite Ceramic Precursor Four-Dimensional Printing

**DOI:** 10.3390/ma19183919

**Published:** 2026-09-15

**Authors:** Xianxiang Gao, Yan Zhu, Jingwen Wu, Junpeng Ma, Peng Qu, Hongshuang Li, Jiayu Dong, Xiangyu Zhu, Anfu Guo

**Affiliations:** School of Mechanical and Automotive Engineering, Liaocheng University, Liaocheng 252000, China; xj2451249646@163.com (X.G.);

**Keywords:** 4D printing, stereolithography, polymer-derived ceramics, molecular dynamics, programmable, self-deploying systems

## Abstract

Four-dimensional (4D) printing is a cutting-edge additive manufacturing technique that enables dynamic deformation of three-dimensional printed components under external stimuli. Polymer-derived ceramics (PDCs) feature excellent design flexibility, corrosion and wear resistance, but ceramic 4D printing still faces critical drawbacks including inferior deformability, low forming precision, poor slurry-photopolymerization compatibility, and single-response actuation limits. This study proposes an innovative strategy for high-resolution, programmable multi-responsive ceramic 4D printing to tackle these issues. We integrated stereolithography (SLA) with thermosetting shape memory polymers (SMPs) and Fe_3_O_4_/Al_2_O_3_ ceramic composites with magnetothermal conversion effects. The prepared composite slurries were systematically characterized, and an integrated process of stepwise curing and shape programming was constructed. Molecular dynamics simulations clarified the interfacial bonding between polymers and inorganic particles. The ceramic precursors achieve autonomous and precise shape recovery under magnetic or thermal stimulation, and the programmed geometries can be stably maintained as designed configurations. This work establishes a complete technical framework that synergistically integrates SLA-based high-precision forming, magnetothermal dual-responsive actuation, and programmable reconfigurability, enabling complex ceramic precursor structures with tunable shape memory effects. The strategy offers a viable route for smart ceramic fabrication and opens promising perspectives for applications in aerospace deployable structures and non-contact biomedical devices.

## 1. Introduction

Additive manufacturing technology has effectively overcome the technical barriers of traditional machining in the fabrication of complex structures, enabling the efficient and customized fabrication of high-precision components. It demonstrates tremendous application potential in high-end scenarios such as aerospace [1], biomedicine [2], and military engineering [3]. As a cutting-edge development direction in the field of additive manufacturing, 4D printing integrates the time dimension and smart responsive materials into the forming system, allowing printed components to achieve dynamic shape evolution under external stimuli [4,5,6], and also providing a novel technical pathway for the intelligent application of ceramic materials.

Polymer-derived ceramics (PDCs) have emerged as a key material system in the field of ceramic additive manufacturing due to their ease of processing, moderate sintering temperatures, and combination of good thermal stability and mechanical properties [7,8]. However, the inherent brittleness and poor deformability of ceramic materials [9,10,11,12] make it difficult to achieve autonomous and controllable dynamic deformation in traditional ceramic 4D printing; this issue has become a core challenge constraining the development of this field [13,14]. Shape memory polymers (SMPs), as typical smart stimulus-responsive materials, are capable of autonomously reverting from a programmed temporary configuration to their initial preset form under external stimuli such as heat [15], light [16,17], electric [18] and humidity [19,20] stimuli. Possessing characteristics such as lightweight, large deformation capacity, and ease of composite processing [21,22,23], they represent an ideal substrate for overcoming the challenges of autonomous deformation in ceramic 4D printing. By compositing SMPs with ceramic precursors, shape memory effects can be imparted to the ceramic precursors, eliminating the reliance of traditional ceramic deformation on external mechanical devices and enabling autonomous, programmable deformation of ceramic structures. However, most existing SMP-composite ceramic systems [24,25,26] can only respond to a single thermal stimulus, lacking the capability for non-contact control, and are thus ill-suited to the demands of dynamic shape control in complex environments. Kong and coworkers [24] developed a VPP-based 4D printing method to manufacture ceramic precursors with reconfigurable and shape memory effects using thermosetting SMPs, yet the system remained limited to single thermal actuation. Similarly, Wu et al. [25] introduced thermoplastic polylactic acid (PLA) into ceramic precursors to achieve resettable permanent shapes through SLA printing, but their approach still relied solely on thermal stimulation and lacked non-contact controllability. These studies indicate that achieving non-contact multi-responsive actuation while maintaining the high-precision forming of SLA remains a significant technical challenge in ceramic 4D printing. It is worth noting that Wu et al. [27] recently reported a magnetothermally hygro-responsive ceramic precursor based on a hydrogel system, where shape changes are driven by dehydration and water absorption, which is fundamentally different from the thermosetting SMP-based shape memory effect employed in the present work in terms of both material system and actuation mechanism.

Fe_3_O_4_ is a spinel-type iron-based magnetic nanoparticle that possesses intrinsic ferromagnetism and highly efficient magnetothermal conversion properties. In an alternating magnetic field, it can rapidly generate heat through hysteresis loss and magnetic moment relaxation, whilst exhibiting excellent chemical stability, thermal stability, and interfacial affinity, without the need for complex surface modification treatments [28]. Existing research has used Fe_3_O_4_ for the 3D printing of complex components [29,30,31,32,33,34] and has further applied it to 4D printing systems [35,36,37,38,39,40]; for instance, Ahangari et al. [40] developed advanced magneto-responsive shape memory nanocomposites embedded with Fe_3_O_4_ nanoparticles, achieving efficient remote magnetic actuation. Inspired by this non-contact strategy, integrating such magnetic-responsive functionalities into ceramic precursor systems offers a promising pathway for intelligent ceramic precursor 4D printing.

However, at the current stage, ceramic 4D printing mostly adopts forming processes such as direct ink writing (DIW) [41,42,43] and material extrusion (ME) [44,45,46]. Restricted by the ink rheological properties and extrusion requirements, it is difficult to meet the standards for forming precision and resolution. Even though relevant studies have realized the autonomous deformation of PDCs through SMP compounding [47,48,49], they are still limited to the aforementioned processes, making it challenging to simultaneously achieve high-precision forming and multifunctional shape memory properties of ceramic structures. As a high-precision additive manufacturing technology [50,51,52], stereolithography (SLA) can fabricate ceramic green bodies with high resolution, excellent dimensional stability, and complete structures through point-by-point laser curing, fundamentally making up for the defects in traditional processes.

To address the research gap in high-resolution, programmable, and multi-responsive ceramic 4D printing, the present study integrates SLA, SMPs, and a Fe_3_O_4_–Al_2_O_3_ ceramic composite system to develop a composite slurry system suitable for SLA fabrication. Employing a stepwise crosslinking and curing strategy, the ceramic precursor is initially shaped via photopolymerization with sufficient flexibility for reconfiguration, and subsequently subjected to thermal curing to stabilize the three-dimensional network. This endows the material with the dual functionalities of shape memory and multi-responsiveness, enabling controlled switching between programmable shaping and precise setting. This technical approach overcomes the key challenges of conventional ceramic 4D printing, namely single-stimulus responsiveness and insufficient shaping precision. The resultant high-resolution, programmable, multi-responsive ceramic 4D printing holds substantial application promise in scenarios such as aerospace self-deployable structures and contactless biomedical devices.

## 2. Experimental Section

### 2.1. Materials

Al_2_O_3_ nanoparticles with an average primary diameter of 100 nm, sourced from Zhongzhi New Materials Co., Ltd. (Jinan, China), were employed in this study. The photosensitive resin (product number: RJ-01) was supplied by King New Materials Co., Ltd., (Shiyan, China). The Fe_3_O_4_ particles (with an average particle size of 20 nm) were purchased from Ruijiang Metal Materials Co., Ltd., (Xingtai, China). The SMPs employed in this study were prepared by compounding bisphenol A diglycidyl ether (DGEBA) and methylhexahydrophthalic anhydride (MHHPA). Among them, the DGEBA epoxy resin monomer was purchased from Olin Corporation, Chesterfield, VA, USA, and the MHHPA curing agent was obtained from Runxiang Chemical Co., Ltd., Changzhou, China.

### 2.2. Preparation of Polymer-Derived Ceramic Precursor (PDCP) Materials

By referring to previous studies [24,47] and conducting extensive pre-experiments, the optimal material ratios and mixing conditions were determined to balance SLA curability, slurry stability, and magnetothermal performance. The preparation process of PDC materials mainly includes the following three steps:

(1) DGEBA and MHHPA were weighed at a mass ratio of 12:10 and fully mixed uniformly to prepare SMPs. Additionally, Al_2_O_3_ particles and Fe_3_O_4_ particles were subjected to vibration sieving treatment to break up powder agglomeration, and then ceramic composite powder was obtained. In addition, each component was weighed separately for later use according to the weight ratio of photosensitive resin:Al_2_O_3_:SMPs:Fe_3_O_4_ = 65:60:22:4.
(2) First, the photosensitive resin was mixed with the SMPs prepared in step (1). A JJ-160 W electric stirrer (Xinrui Instrument Factory, Xicheng, Jintan District, Changzhou, China) was used to stir at a rotating speed of 1800 r/min for 5 min. During the entire process, ultrasonic vibration and heating were employed to fully homogenize the photosensitive resin and SMPs, so as to obtain photosensitive resin premixed slurry.
(3) The standby Al_2_O_3_ powder and Fe_3_O_4_ powder were added to the photosensitive resin premixed slurry obtained in step (2), and stirring was continued at a rotating speed of 1800 r/min for 25 min to ensure that the ceramic particles and magnetic nanoparticles were uniformly dispersed in the photosensitive resin system without agglomeration. The whole process was also assisted by ultrasonic vibration and heating. Finally, the mixed slurry was degassed in a vacuum environment to remove bubbles and pores in the slurry, and the composite ceramic slurry applicable for SLA printing was obtained.

### 2.3. Additive Manufacturing

In this study, SLA technology was selected for the additive manufacturing of PDC materials. The printing equipment used in the experiment was an AME RP150 stereolithography printer, which was developed and produced by Shanghai UnionTech Co., Ltd., (Shanghai, China), which is suitable for the high-precision photocuring of high-precision polymer-derived ceramic precursor (PDCP) slurries for fabricating shape memory polymers (SMPs). For the process used in preparing SLA-printed green bodies, this study systematically reviewed and synthesized previous research results and relevant authoritative literature in the field. Combined with multiple sets of pre-experiment debugging, parameter comparison, and iterative optimization under the experimental procedure, the optimal forming-process parameters suitable for this study’s SMPs–PDCP system were finally selected and determined.

The specific core process parameters were set as follows: the single-layer printing thickness was maintained at 0.05 mm, the laser curing power was set at 266 mW, the laser fillings were performed 8 times, and the laser scanning spacing was 0.03 mm. The internal filling of the green body adopted the X–Y staggered mode, and the scraper coating process was set to two reciprocating scrapings to ensure that the ceramic precursor slurry was evenly spread on the printing platform without bubble residue and that the photocuring reaction was sufficient. The results of a large number of pre-experiments confirmed that these regulated process parameters could effectively avoid obvious structural defects in the Fe_3_O_4_-SMPs-PDCP formed during SLA printing, and ensure the structural integrity and the forming stability of the printed green bodies.

### 2.4. Structural Programming Design of PDCP

During the SLA additive manufacturing process of the Fe_3_O_4_-doped PDC composite system, the photosensitive resin in the slurry is converted from a liquid state to a solid state with an initial configuration through a laser-triggered crosslinking polymerization reaction, which process is defined as primary curing. After primary curing, the Fe_3_O_4_-containing SMPs-PDCP still maintains excellent flexibility, and the as-printed geometry can be precisely adjusted using either molds or fixtures [53,54]. For the regulated configuration, two curing paths can be adopted to achieve shaping: first, it is subjected to secondary curing at 150 °C for 2 h, so that Fe_3_O_4_ forms a stable crosslinked network with the polymer matrix and ceramic particles. Even if the mold or fixture is removed, the SMPs-PDCP can still maintain the regulated target configuration persistently; second, if the secondary curing temperature is set to 120 °C, the SMPs-PDCP can be temporarily reconstructed into other preset shapes. When the temperature decreases to ambient temperature, the system’s molecular chains become immobilized, and this transient geometry remains fully fixed and stable following stress relaxation; upon reheating to 100 °C, the temporary shape will spontaneously revert to the target configuration defined during secondary curing.

### 2.5. Measurements and Tests

To systematically assess the suitability of PDC materials for SLA printing and characterize their performance, rheological behavior analysis and printability verification were conducted with a microvisual rotational rheometer (MCR302, Anton Paar GmbH, Graz, Austria); a Fourier-transform infrared (FTIR) spectrometer (Thermo Fisher Scientific Nicolet iS20, USA) was utilized to elucidate the crosslinking reaction process; a dynamic mechanical analyzer (Dynamic thermomechanical analysis (DMA), TA Q800, USA) was used to determine the shape-memory transition temperature; scanning electron microscopy (SEM) coupled with energy dispersive X-ray spectroscopy (EDS) (ZEISS Sigma 360, Germany) was used for micromorphology observation and elemental analysis. For the evaluation of forming precision, the VMA 2515 image-based dimensional measurement instrument (Tianzhun Technology Co., Ltd., Suzhou, China) was employed; the alternating magnetic field required for magnetothermal actuation was generated by a high-frequency induction heating device (HDC-30 KW, Shanghai Honghu Electromechanical Equipment Co., Ltd., Shanghai, China); finally, an electronic universal testing machine (JD3310W, Cangzhou Yixuan Experimental Instrument Co., Ltd., China) was used in strict accordance with the GB/T 23805-2006 [55] standard.

Five parallel samples were tested under each set of conditions, and the test force and displacement data of each specimen were collected. Subsequently, the stress–strain curves were derived based on Formulas (1) and (2) [56,57], where the physical quantities are defined as follows: *F* is the applied force, *A* denotes the initial cross-sectional area of the specimen, *σ* represents the stress, *ε* is the strain, Δ*L* indicates the displacement, and *L*_0_ is the original length of the specimen. Data processing and plotting were performed using Origin (version 2024, OriginLab Corporation, Northampton, MA, USA).

(1) $$\sigma =\frac{F}{A}$$

(2) $$\varepsilon =\frac{\Delta L}{L_{0}}$$

## 3. Results and Discussion

### 3.1. Synthesis of Fe_3_O_4_-SMPs-PDCP

**Figure 1:**
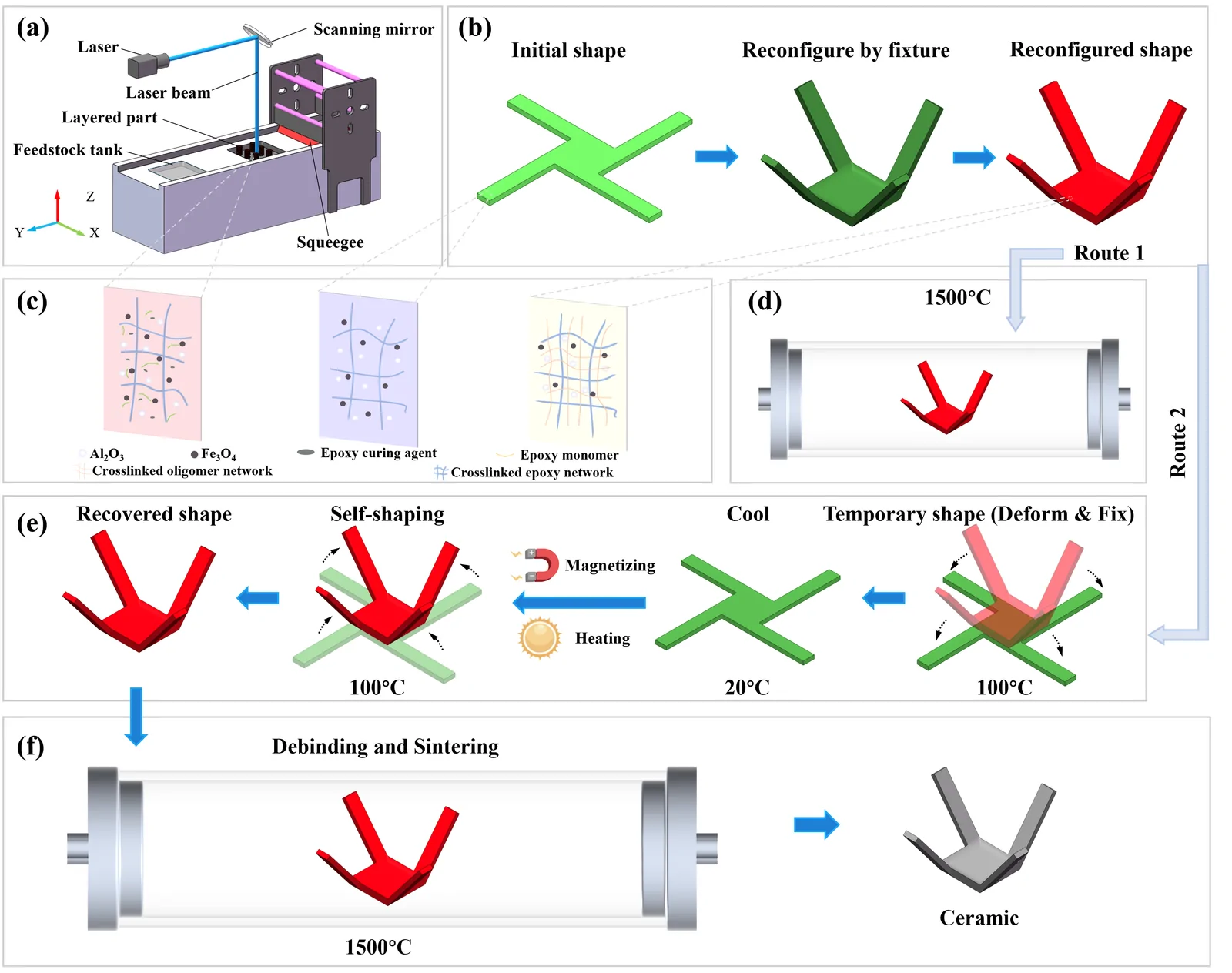
Schematic illustration of Fe_3_O_4_-SMPs-PDCP fabrication under the reconfigurable and programmable SLA technique: (**a**) SLA additive manufacturing workflow; (**b**) shape reconfiguration mechanism of Fe_3_O_4_-SMPs-PDCP; (**c**) network evolution of Fe_3_O_4_-SMPs-PDCP throughout the two-step curing process; (**d**) Route 1 for Fe_3_O_4_-SMPs-PDCP: one-step pyrolysis to achieve ceramicization; (**e**) Route 2 for Fe_3_O_4_-SMPs-PDCP: morphological transition from temporary state to recovered configuration; (**f**) ceramic fabrication via pyrolysis treatment.

Figure 1 is a schematic diagram illustrating the principle of the reconfigurable and programmable Fe_3_O_4_-SMPs–PDCP prepared based on SLA technology. This study innovatively incorporated Fe_3_O_4_ nanoparticles into the PDC material system, enabling the uniform dispersion of Fe_3_O_4_ nanoparticles and endowing the material with magnetic response functionality. During the initial printing stage, the scraper uniformly coats the Fe_3_O_4_-containing PDC composite slurry on the forming platform, and the laser above the platform scans the slurry according to a preset path to trigger the curing reaction, as shown in Figure 1a. Subsequently, the forming platform descends by one layer thickness, and the coating and curing processes are repeated to complete the green body preparation through layer-by-layer superposition, as illustrated in Figure 1a. Figure 1b illustrates the reconfiguration mechanism of the as-printed Fe_3_O_4_-SMPs–PDCP: after the first curing step, the precursor retains high ductility and develops a partially crosslinked polymeric network, as shown in Figure 1c. This property enables shaping through molds, fixtures, or origami processes; subsequent secondary curing is performed at 150 °C for 2 h, enabling the precursor to form a fully crosslinked network, and the adjusted geometry is stably locked as the target configuration. This target configuration can be directly transformed into ceramic materials via thermal degreasing and high-temperature sintering, corresponding to the Route 1 pathway depicted in Figure 1d.

SMPs-PDCP containing Fe_3_O_4_- exhibits a significant thermally induced shape memory effect and an additional magnetic response characteristic: the precursor after the second curing step can be reprogrammed into any temporary shape at 100 °C, a temperature higher than the transition temperature (T_trans_). When cooled to room temperature (lower than T_trans_), the molecular chains inside the precursor freeze, and the temporary shape can remain intact even after the external force is unloaded, which is beneficial for optimizing spatial storage. When thermal stimulation or external magnetic field stimulation is applied again, the temporary shape can autonomously recover to the target configuration. The magnetic response function provides a dual thermomagnetic response pathway for shape regulation, as shown in Figure 1e. In addition, by integrating the shape recovery program with the sintering process, continuous and controllable transformation of the precursor shape can be achieved during a single initial heating process, while completing the conversion from polymer to ceramic, corresponding to Route 2 shown in Figure 1f. Currently, most SMP ceramic materials can only be reconfigured in response to a single stimulus, which limits their application scenarios. In this study, through the introduction of Fe_3_O_4_ and optimization of the material system, reconfigurability and programmability are achieved, and the magnetic response function is expanded, significantly enhancing the application potential of the material in scenarios such as intelligent manufacturing and multifunctional response.

### 3.2. Material Design and Validation of Additively Manufactured Fe_3_O_4_-SMPs–PDCP

**Figure 2:**
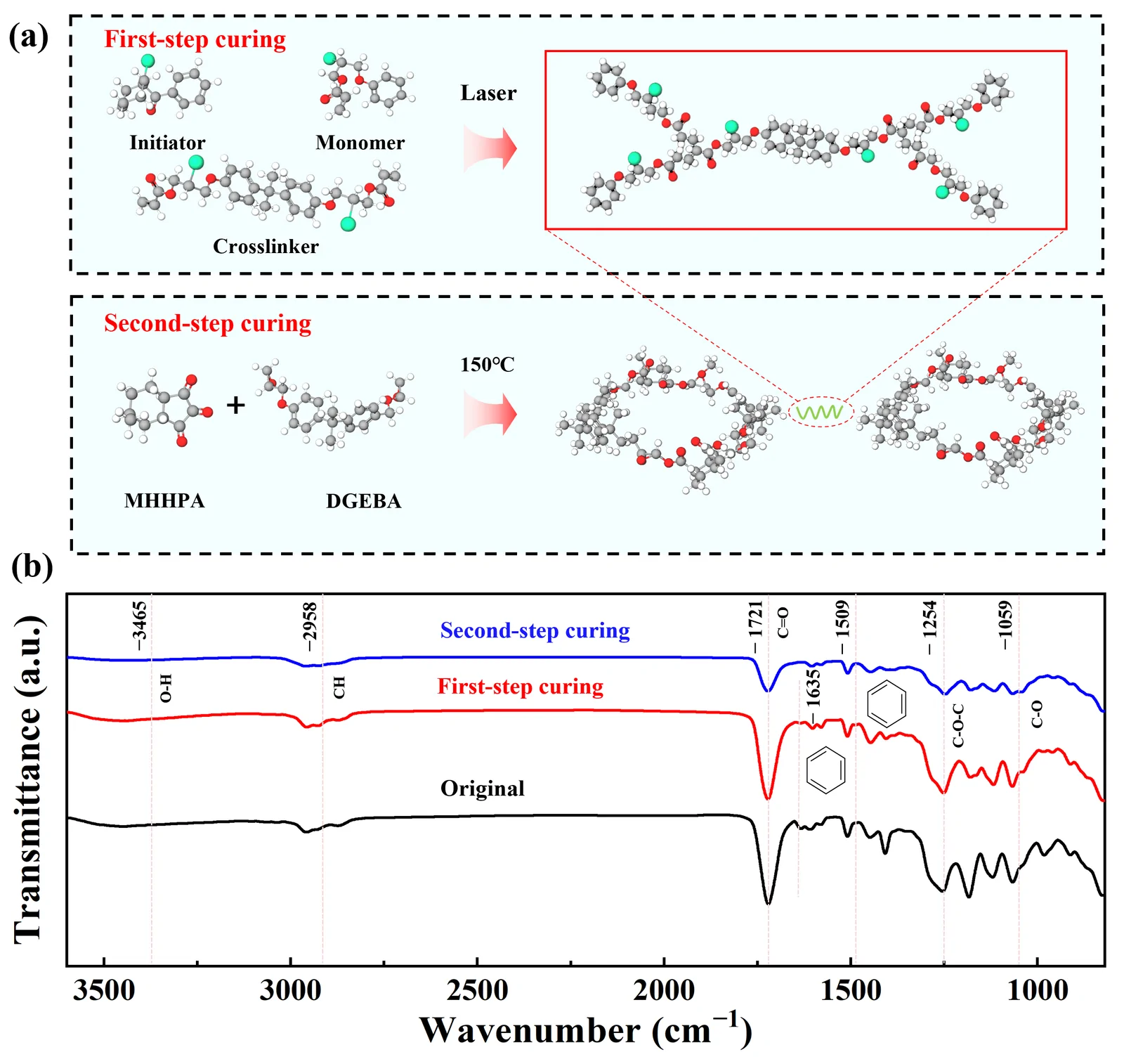
Synthesis process and performance characterization of Fe_3_O_4_-SMPs-PDCP: (**a**) schematic illustration of the two-step curing mechanism detailing the chemical structures and reaction pathways; (**b**) FTIR spectra of Fe_3_O_4_-SMPs-PDCP samples in the uncured, first-stage cured, and second-stage cured states.

Material design is crucial for the additive manufacturing of ceramic components with complex geometries. As shown in Figure 2a, this system is constructed using a two-step curing strategy. In the initial photocuring stage, the photoinitiator is excited from the ground state to the excited state and releases active free radicals, which subsequently initiate the chain polymerization of monomers containing carbon–carbon double bonds or cyclic structures. With the gradual formation of covalent bonds, a partially crosslinked network structure is established, realizing the transition of the slurry from a liquid to a solid state. As shown in Figure 2b, FTIR analysis further confirms this process, which is consistent with the preliminary photocuring pathway utilizing photosensitive resin polymerization reported by Kong et al. [24] and the network construction pathway via anhydride–epoxy ring-opening and esterification during thermal curing described by Chen et al. [47]. Specifically, the raw material exhibits a C=C absorption peak at 1635 cm^−1^. After initial photocuring, this peak is significantly attenuated, while the C=O peak at 1721 cm^−1^ and other characteristic peaks remain unchanged, confirming the partial crosslinking of the resin and providing the green body with the flexibility required for reconfiguration. Upon secondary thermal curing at 150 °C, in line with the ring-opening and esterification pathways detailed by Chen et al. [47], the C=C signal at 1635 cm^−1^ completely disappears, and characteristic peaks within the 1254 to 1066 cm^−1^ range shift, indicating the formation of an interpenetrating polymer network structure. This controlled transition from partial to full crosslinking establishes the robust network foundation essential for elastic potential energy storage and the shape memory effect.

**Figure 3:**
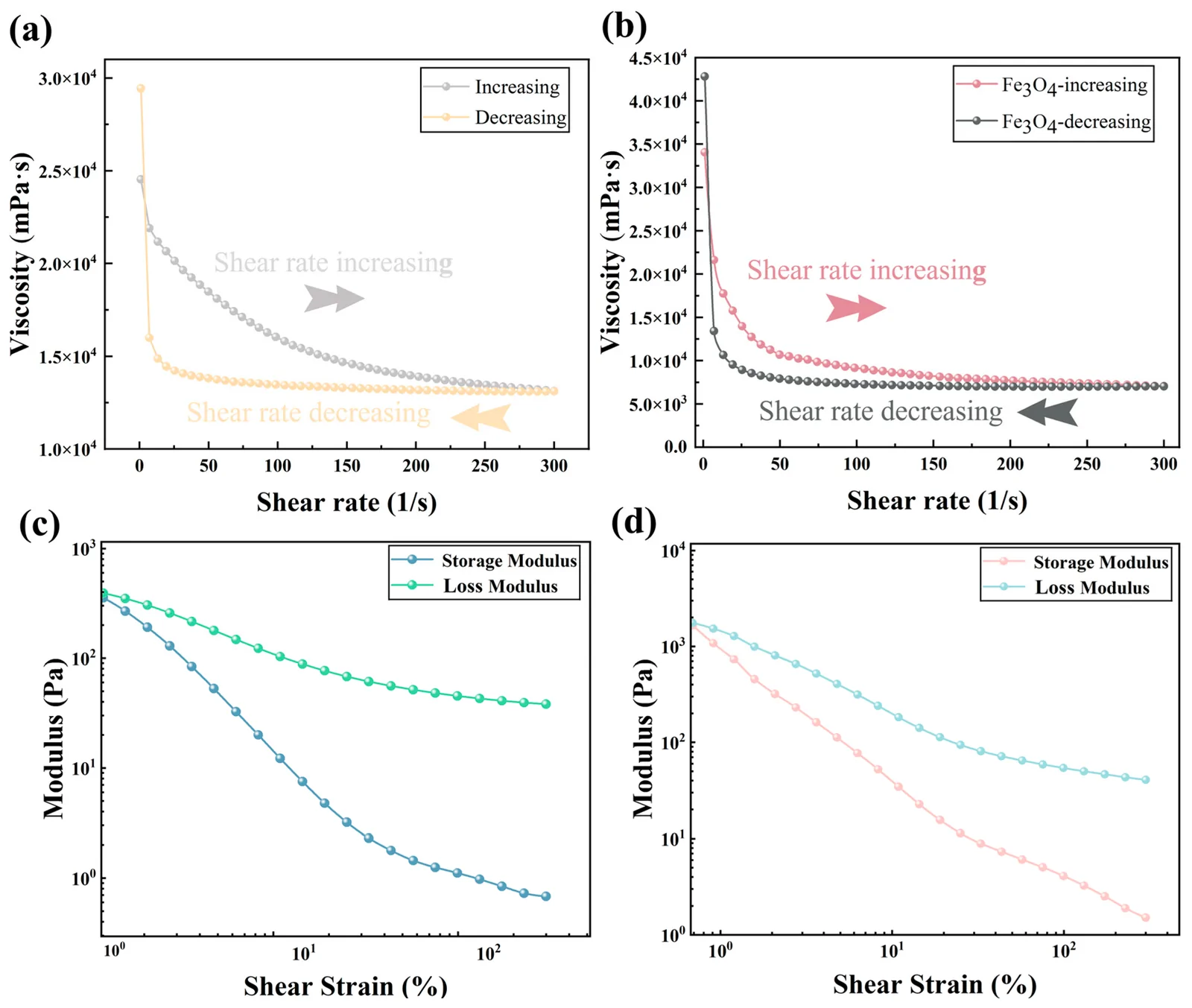
(**a**,**b**) Variations in viscosity as a function of increasing and decreasing shear rate for pristine SMPs-PDCP and Fe_3_O_4_-modified SMPs-PDCP composites. (**c**,**d**) Evolution of storage modulus and loss modulus versus shear strain for SMPs-PDCP and Fe_3_O_4_-SMPs-PDCP materials.

Material development is core to the success of SLA additive manufacturing, and its rheological properties directly determine the forming quality and precision of printed structures. Since the wiping mechanism requires reciprocating motion during the printing process to achieve uniform slurry spreading, the material must satisfy three core requirements: uniform dispersion, shear response adaptability, and shape retention capability. Figure 3a,b show the viscosity versus shear rate curves including increasing and decreasing processes, and Figure 3c,d show the viscoelastic property curves, analyzed in combination with the introduction of Fe_3_O_4_. Among them, Figure 3a,c represent the control group without Fe_3_O_4_, and Figure 3b,d represent the experimental group with Fe_3_O_4_ added.

Both groups of slurries exhibit a shear-thinning effect, with viscosity decreasing as the shear rate increases, confirming their non-Newtonian fluid characteristics [58,59,60], which complies with the requirements for shear response and uniform dispersion. To further reveal their rheological behavior, curves at decreasing shear rates were introduced for comparison. As shown in Figure 3a,b, during the decreasing shear rate stage, the viscosity curves of the slurries are globally lower than those at increasing shear rates, forming obvious thixotropic loops or hysteresis loops. This phenomenon originates from the regulation and disruption of the internal particle aggregation structures of the slurries by shear force: under low shear conditions, weak interactions among Al_2_O_3_ particles form a stable network, leading to high viscosity; as the shear rate increases, the network is gradually destroyed, particle dispersion is improved, and viscosity decreases significantly. After adding Fe_3_O_4_, as shown in Figure 3b, the thixotropic trend remains consistent, but under low-shear conditions, due to magnetic dipole interactions and adsorption between particles and the resin, the overall viscosity increases slightly; under high shear, this structure can still be effectively disintegrated, keeping the viscosity within a suitable range compatible with printing. This thixotropy enables the slurries to rapidly reduce viscosity for leveling during blade spreading, and quickly recover local structures after shear ceases, which is beneficial for maintaining shape.

In addition, as shown in Figure 3c,d, the loss modulus consistently exceeds the storage modulus under the corresponding shear strain, revealing the viscoelasticity of the PDC materials [61,62,63]. In the reference group, as shown in Figure 3c, the structure remains stable during the early stage of shear strain variation, which is conducive to forming a uniform coating and resisting deformation, and exhibits good late-stage flow adaptability to achieve a viscoelastic balance. After incorporating Fe_3_O_4_, as shown in Figure 3d, the slurry possesses better shape retention and deformation resistance, with superior structural stability in the low strain range compared with the control group, and the overall flow adaptability is not impaired even under high strain. In summary, both slurries meet the rheological requirements of SLA printing, and the introduction of Fe_3_O_4_ moderately adjusts viscosity, thixotropy, and structural stability through interparticle interactions, providing key rheological support for the additive manufacturing of magnetically responsive smart materials.

### 3.3. Properties of Additively Manufactured Fe_3_O_4_-SMPs–PDCP

**Figure 4:**
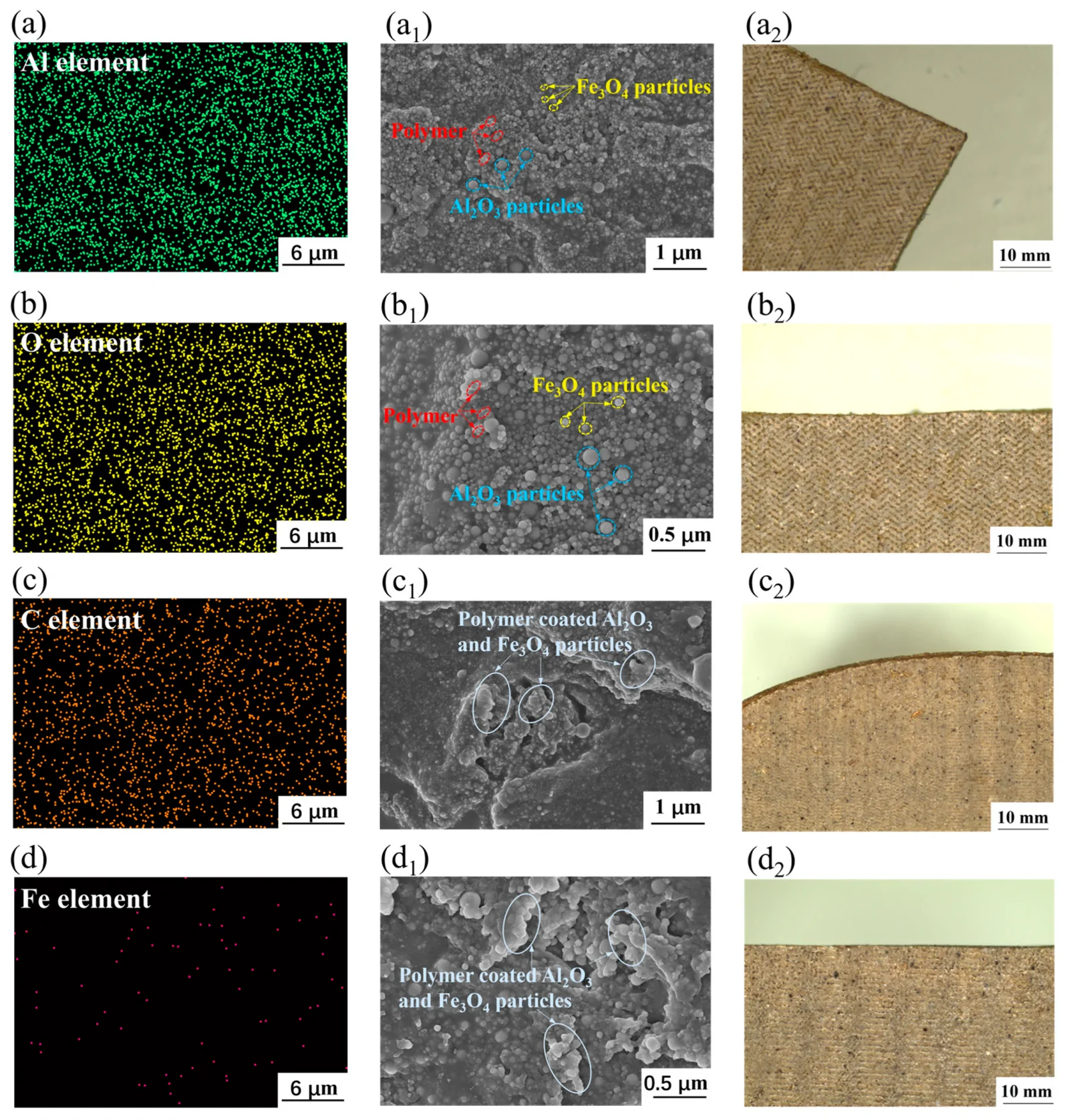
Microstructure of ceramic precursors. (**a**–**d**) SEM images showing the EDS images of Fe_3_O_4_-SMPs-PDCP elements; (**a_1_**,**b_1_**) microstructure after initial curing; (**c_1_**,**d_1_**) microstructure following secondary curing; (**a_2_**,**b_2_**) optical microscopy images of the surface features of ceramic precursors after initial curing; (**c_2_**,**d_2_**) optical microscopy images of the surface features of ceramic precursors following secondary curing.

To clarify the regulatory characteristics of the two-step curing reaction on the surface morphology and microstructure of the composite ceramic precursor, EDS detection was performed on SMPs-PDCP, as shown in Figure 4a–d. SEM characterization was also conducted on the samples at different curing stages. The composite ceramic slurry was converted into a ceramic precursor through the first-step curing, during which the photosensitive resin underwent photopolymerization and curing under laser induction, resulting in an interwoven distribution of the polymer phase, Al_2_O_3_ particles, and Fe_3_O_4_ particles, as illustrated in Figure 4(a_1_,b_1_).

Under high-temperature conditions, the ceramic precursor initiates the second-step curing process, where SMPs undergo crosslinking reactions, realizing the tight coating of the polymer phase on Al_2_O_3_ particles and Fe_3_O_4_ particles. This process constructs a continuous 3D composite network structure of polymer and inorganic particles, as shown in Figure 4(c_1_,d_1_). In addition, it can be clearly observed from the SEM image characterization that the polymer phase, Al_2_O_3_ particles, and Fe_3_O_4_ particles are all uniformly dispersed without obvious agglomeration or segregation.

An optical microscope was used to characterize and compare the surface morphology of Fe_3_O_4_-SMPs–PDCP under the two-step curing process. Among them, Figure 4(a_2_,b_2_) show the surface morphology of the Fe_3_O_4_-SMPs–PDCP after the first-step curing; at this stage, the polymer matrix has completed preliminary crosslinking, forming a prepolymerized network that supports inorganic particles. Figure 4(c_2_,d_2_) correspond to the surface morphology of the ceramic precursor after the second-step curing; at this stage, the SMP component undergoes further reactions, constructing a dense 3D crosslinked network. The microscopic surface texture observed in these images is primarily attributed to the subtle laser raster patterns from stereolithography printing combined with the optical diffuse reflection on the densely distributed inorganic nanoparticles, rather than physical voids or porosity. Comparative analysis of the morphology images confirms that the precursor samples at both curing stages maintain intact, compact structures without obvious microdefects or internal cavities.

**Figure 5:**
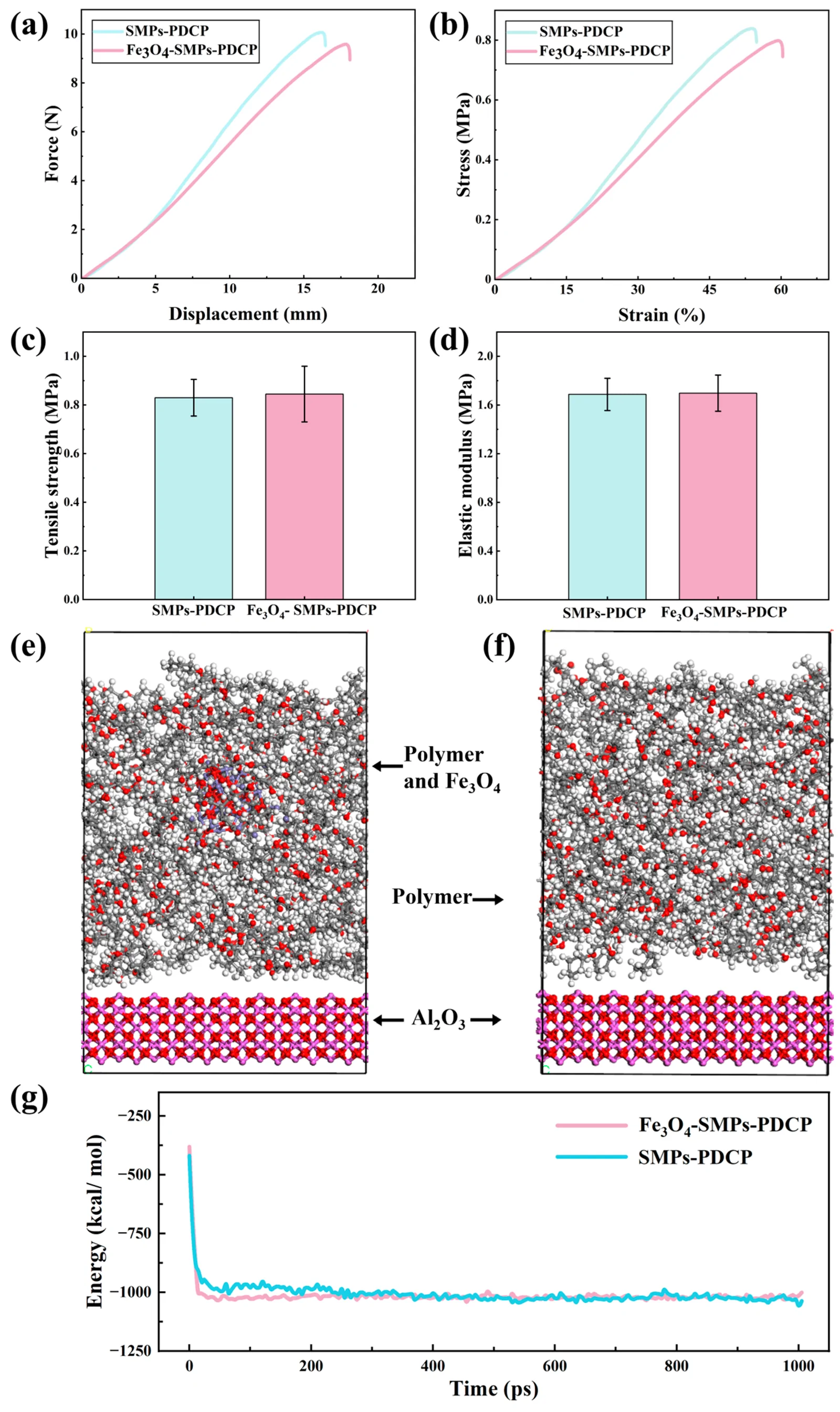
Mechanical tests and MD simulations of Fe_3_O_4_-SMPs–PDCP and pristine SMPs-PDCP. (**a**) Force–displacement curves of different specimens; (**b**) stress–strain curves of different specimens; (**c**) comparison of tensile strength for different specimens; (**d**) comparison of elastic modulus for different specimens; (**e**,**f**) interfacial structures of Fe_3_O_4_-SMPs–PDCP and SMPs-PDCP; (**g**) comparison of binding energy between Fe_3_O_4_-SMPs–PDCP and SMPs-PDCP.

To investigate the influence of Fe_3_O_4_ nanoparticle incorporation on the mechanical properties of Fe_3_O_4_-SMPs–PDCP, uniaxial tensile tests were conducted with pure SMPs-PDCP (without Fe_3_O_4_ addition) as the reference specimen and Fe_3_O_4_-SMPs–PDCP as the composite specimen. The corresponding mechanical property curves and statistical results are displayed in Figure 5a–d.

As depicted in Figure 5a,b, both specimen groups exhibit the typical elastoplastic deformation behavior of polymer–matrix composites. At the initial loading stage, an approximately linear elastic deformation regime is observed, where the force and stress increase synchronously with displacement and strain. Subsequently, the specimens enter the plastic deformation stage, accompanied by a gradual decrease in the curve slope until final fracture occurs. The SMPs-PDCP reference group only shows a slight superiority in peak bearing capacity and peak stress, with consistent mechanical evolution trends and a small numerical difference between the two groups.

The quantitative mechanical results presented in Figure 5c,d further corroborate the regularity described above. Compared with the SMPs-PDCP specimen, the Fe_3_O_4_-SMPs–PDCP composite exhibits only a marginal enhancement in elastic modulus and tensile strength. The differences between the two datasets are negligible, and their error bar ranges largely overlap, demonstrating that the incorporation of Fe_3_O_4_ nanoparticles exerts no adverse effect on the mechanical stability of the material system, but instead produces a slight reinforcing effect on the stiffness and strength of the composites.

Notably, the pristine SMPs-PDCP without Fe_3_O_4_ incorporation appears as a light-colored printed part with relatively low forming difficulty during SLA fabrication. In contrast, the Fe_3_O_4_-SMPs–PDCP composite with Fe_3_O_4_ addition presents a dark-colored printed part, and its forming difficulty is considerably increased due to the light-absorption characteristics of the composite material.

Despite the higher forming difficulty of the dark-colored printed components, both specimen groups still maintain comparable levels of mechanical properties. This finding demonstrates that the optimized SLA printing process developed in this study plays a critical role in guaranteeing the forming quality of components and the stability of their mechanical performance, verifying the effectiveness and practicality of improvements in the process.

To verify the macroscopic mechanical experimental results at the molecular level and reveal the mechanism by which Fe_3_O_4_ incorporation affects the microscopic interface and mechanical behavior of the material, comparative analyses were further conducted using molecular dynamics (MD) simulations performed in BIOVIA Materials Studio (version 2023, Dassault Systèmes, San Diego, CA, USA) on both the SMPs-PDCP and Fe_3_O_4_-SMPs–PDCP systems (Figure 5e,f). The simulation results are consistent with the conclusions derived from macroscopic mechanical tests, which fully validates the aforementioned mechanical performance regularity. The minor discrepancies in interfacial binding energy (Figure 5g) and mechanical properties between the Fe_3_O_4_-SMPs–PDCP and SMPs-PDCP systems provide a microscopic theoretical explanation for the experimentally observed similarity in their mechanical properties.

**Figure 6:**
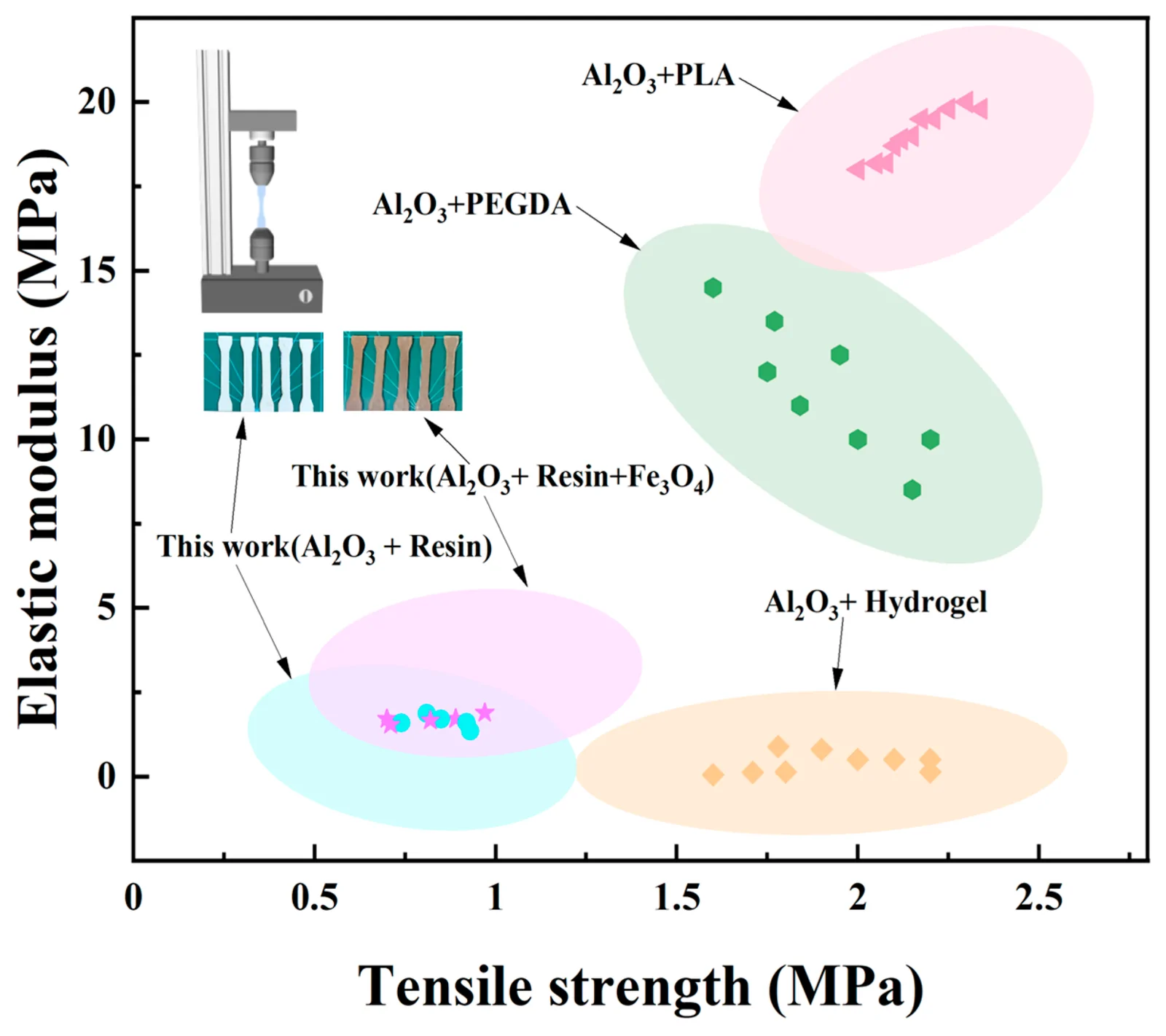
Tensile property comparison: elastic modulus and tensile strength of this work versus data from references [26,32,64].

The Ashby plot comparison in Figure 6 demonstrates that the Fe_3_O_4_-SMPs–PDCP composite precursor prepared in this study presents balanced performance in elastic modulus and tensile strength, with its mechanical properties falling within a reasonable range similar to most flexible ceramic-based materials. The moderate modulus endows the material with sufficient flexible deformability to satisfy the mechanical demands of repeated deformation during magnetic actuation.

Overall, the incorporation of Fe_3_O_4_ slightly elevates the elastic modulus and tensile strength of the material while maintaining a high degree of mechanical similarity to SMPs-PDCP. Furthermore, it optimizes the plasticity and ductility of the material and simultaneously imparts magnetic responsiveness to the material, achieving the synergistic optimization of mechanical properties and functional characteristics. This lays a solid foundation for the preparation and application of magnetically responsive smart ceramics.

### 3.4. Shape Memory Representation

Two comparative groups, namely SMPs-PDCP and Fe_3_O_4_-SMPs-PDCP, were established in this study to evaluate and compare their shape memory performance. At the initial experimental stage, rectangular specimens with dimensions of 1 mm × 4 mm × 30 mm were subjected to bending deformation at 100 °C, and the corresponding fixed angle θ_1_ was meticulously documented. After cooling the specimens to a room temperature of 22 °C, they were reheated to 100 °C, during which the shape recovery angle θ_2_ was simultaneously measured and recorded. The shape fixation ratio, R_f_, and shape recovery ratio, R_r_, were calculated using Equations (3) and (4).

(3) $$R_{f}=(1-\frac{\theta_{1}}{180{}^{\circ}})\times 100\%$$

(4) $$R_{r}=(1-\frac{\theta_{2}}{180{}^{\circ}-\theta_{1}})\times 100\%$$

**Figure 7:**
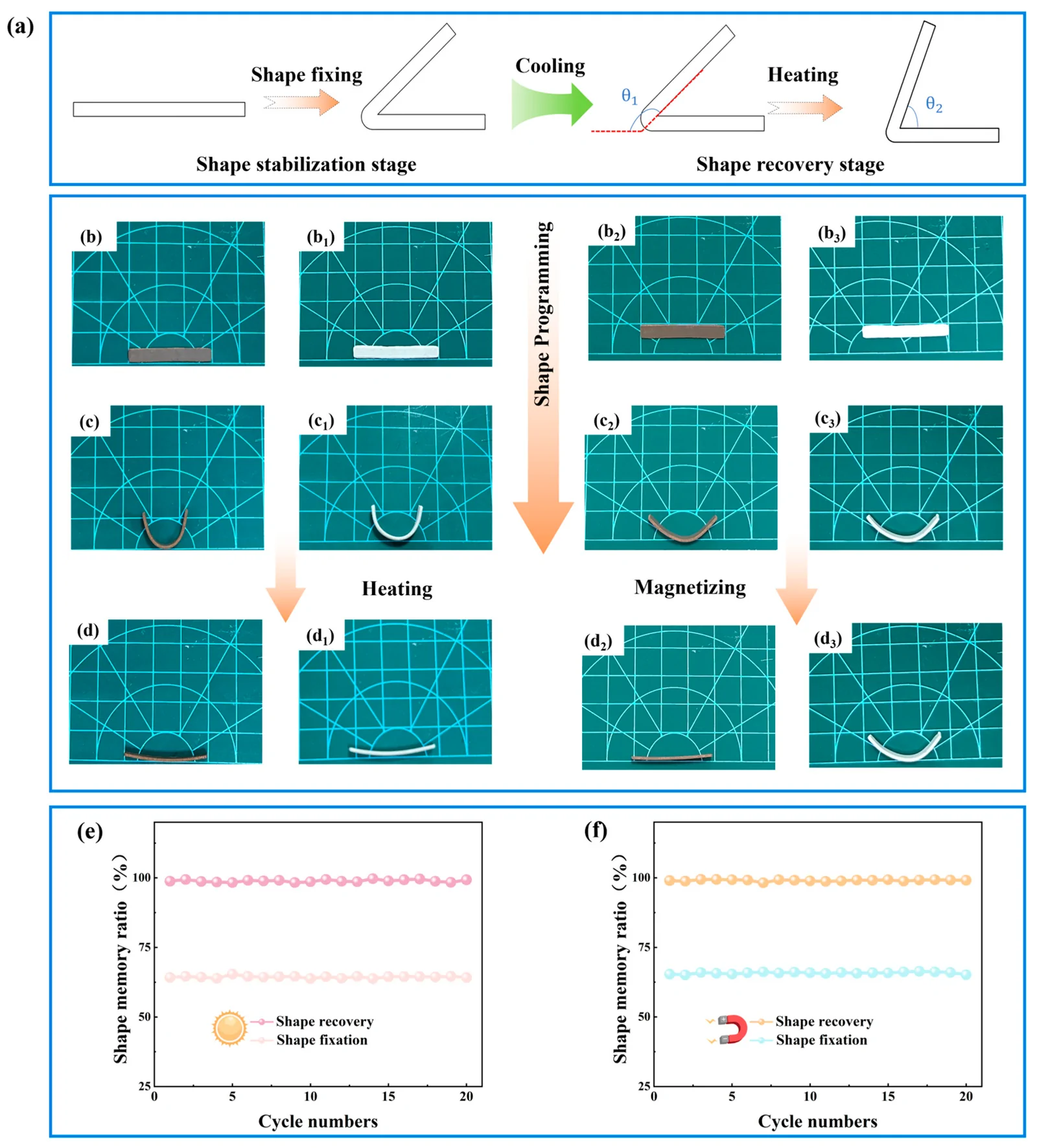
Comparison of shape memory functions and cycle performance between programmed SMPs-PDCP and Fe_3_O_4_-SMPs-PDCP under thermal and magnetic stimulations. (**a**) quantification method; (**b**) programmed shapes; (**c**) fixed temporary shapes; (**d**) shape recovery under thermal and magnetic stimulations; (**e**) cyclic performance under thermal stimulation; (**f**) cyclic performance under magnetic actuation.

To investigate the multifunctional programmable properties of Fe_3_O_4_-SMPs-PDCP, the two sample groups were selected for comparative tests on shape memory behavior under direct heating and magnetic field application. Figure 7 illustrates the shape memory function from the perspective of both angle and morphological changes under different stimulations. Specifically, Figure 7a demonstrates the quantification method of the shape memory performance. The initial shapes after programming at 100 °C are displayed in Figure 7(b,b_1_–b_3_), while the final set shapes after cooling are exhibited in Figure 7(c,c_1_–c_3_). Upon secondary heating, the cooled samples successfully recovered to the shapes shown in Figure 7(d,d_1_), indicating that both SMPs-PDCP and Fe_3_O_4_-SMPs-PDCP possess excellent thermally responsive programmable shape memory properties. However, the states of the samples after applying a magnetic field reveal a stark contrast. As seen in Figure 7(d_2_,d_3_), the pristine SMPs-PDCP failed to maintain the pre-programmed shape under the action of the magnetic field, indicating that it lacks programmability under magnetic actuation. In contrast, the Fe_3_O_4_-SMPs-PDCP successfully memorized and recovered the programmed shape, demonstrating that this composite material possesses magnetothermally dual-responsive shape memory characteristics.

To more accurately quantify the shape-memory recovery capability, Figure 7e,f depict the shape-memory recovery performance of Fe_3_O_4_-SMPs-PDCP under thermal stimulation and magnetic field stimulation, respectively, over 20 consecutive cycles. During these cyclic tests, the material exhibited highly stable shape memory characteristics in both stimulation modes. The shape fixity ratio, *R_f_*, was maintained at approximately 65%, and the shape recovery ratio, *R_r_*, consistently reached about 99% in both cases. This excellent reproducibility indicates that the ceramic precursors can be effectively reprogrammed into various temporary configurations and reliably recover their original shapes.

### 3.5. Additive Manufacturing of Complex Fe_3_O_4_-SMPs–PDCP

To systematically verify the magnetothermal dual-response shape memory efficiency of Fe_3_O_4_–SMPs–PDCP, SLA additive manufacturing technology was employed to print various complex structural models, including manipulators, dragonflies, and petals. The alternating magnetic field for magnetothermal actuation was generated by a high-frequency induction heating device (HDC-30 KW, Shanghai Honghu Electrical Equipment Co., Ltd.) with an operating frequency of 50 kHz and the analog current dial set to the maximum position (reading 1150); the induction coil was water-cooled with a cooling water pressure above 0.2 MPa and a flow rate above 6 L/min to ensure stable operation. Deformation performance tests were conducted through two pathways (magnetic stimulation and thermal stimulation) to comprehensively explore the programmable deformation potential and structural stability of the material.

**Figure 8:**
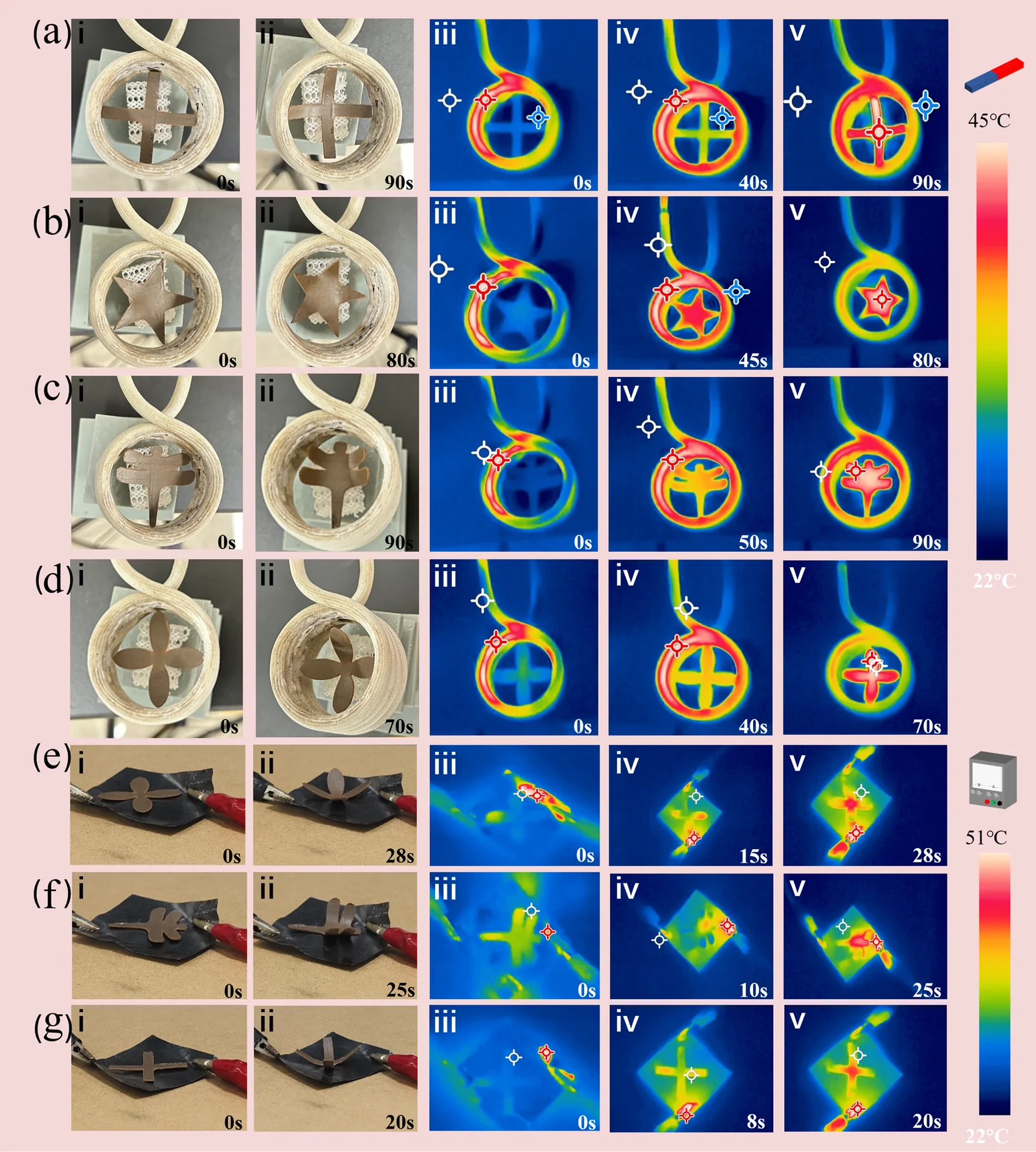
Shape memory changes in Fe_3_O_4_-SMPs–PDCP with complex shapes: (**a**–**d**) magnetic field-actuated shape recovery; (**e**–**g**) thermally induced shape recovery; (**i**) initial printed shape; (**ii**) final set shape. (**iii**–**v**) Thermal imaging sequence corresponding to the deformation process: (**iii**) initial temperature field; (**iv**) intermediate deformation stage of Fe_3_O_4_ magnetothermal heating under magnetic field excitation; (**v**) temperature distribution and final configuration after shape recovery.

To verify the magnetic response deformation, Figure 8a–d use a magnetic field as a non-contact driving medium, realizing precise regulation of structural deformation based on the magnetic response function of Fe_3_O_4_ nanoparticles. All samples were initially printed in a planar shape, a design that can minimize raw material consumption and shorten the preparation cycle of complex configurations. Taking advantage of the excellent flexibility of Fe_3_O_4_-SMPs–PDCP, the planar samples were shaped into preset reconfigurable shapes (target shapes) such as the clamping state of a manipulator, the blooming state of petals, and the spreading state of dragonfly wings through custom molds. Subsequently, the samples were further reconfigured into temporary shapes. When the samples were placed in a magnetic field generated by a coil, the Fe_3_O_4_ particles were subjected to magnetic field action to induce interfacial stress transfer, which drove the samples to autonomously and rapidly recover from the temporary shapes to the preset target shapes. The deformation process was stable and orderly, without defects such as structural cracking, delamination, or fracture. Complex models including manipulators, dragonflies, and petals all exhibited reliable magnetically controlled deformation capabilities, which fully confirmed that Fe_3_O_4_-SMPs–PDCP has precise shape-memory regulation effects under magnetic field stimulation.

To verify the thermally responsive deformation, Figure 8e–g adopt graphene paper electrothermal heating as the triggering method, realizing structural deformation relying on the intrinsic thermally induced shape memory effect of the material. Similarly, all samples were initially printed in a planar shape. After being fixed into reconfigurable shapes (such as petal-blooming state, manipulator-clamping state, and dragonfly-wing-spreading state) by molds, they were further reconfigured into temporary shapes. The precursor samples were attached to graphene paper, and after energizing the graphene paper through a DC power supply, it rapidly generates heat and conducts the heat uniformly to the interior of the precursor. This triggers the thermally induced shape memory effect of Fe_3_O_4_-SMPs–PDCP: under the action of heat, the polymer molecular chains regain mobility, driving the sample to autonomously unfold from the temporary shape and accurately recover to the preset target shape. The thermal response processes of the petal, dragonfly, and manipulator models are efficient and controllable, and no structural damage caused by stress concentration occurred during the deformation process, demonstrating excellent thermal response stability and fracture resistance.

These experimental results indicate that the complex structures of Fe_3_O_4_-SMPs–PDCP printed by SLA technology can achieve active transformation and dynamic shape adjustment between preset configurations under the action of magnetic stimulation or thermal stimulation. The shape evolution process exhibits significant time-dependent characteristics, confirming that this material system possesses efficient 4D printing performance. Under the magnetothermal dual-response pathway, the material exhibits excellent shape memory properties. Moreover, it has good compatibility with the SLA additive manufacturing process, and integrates programmability and reconfigurability. This provides key technical support for the application of dynamic ceramic structures in multiple scenarios such as non-contact regulation and contact-based precise temperature control.

### 3.6. Dynamic Thermomechanical Properties of Fe_3_O_4_-SMPs–PDCP

**Figure 9:**
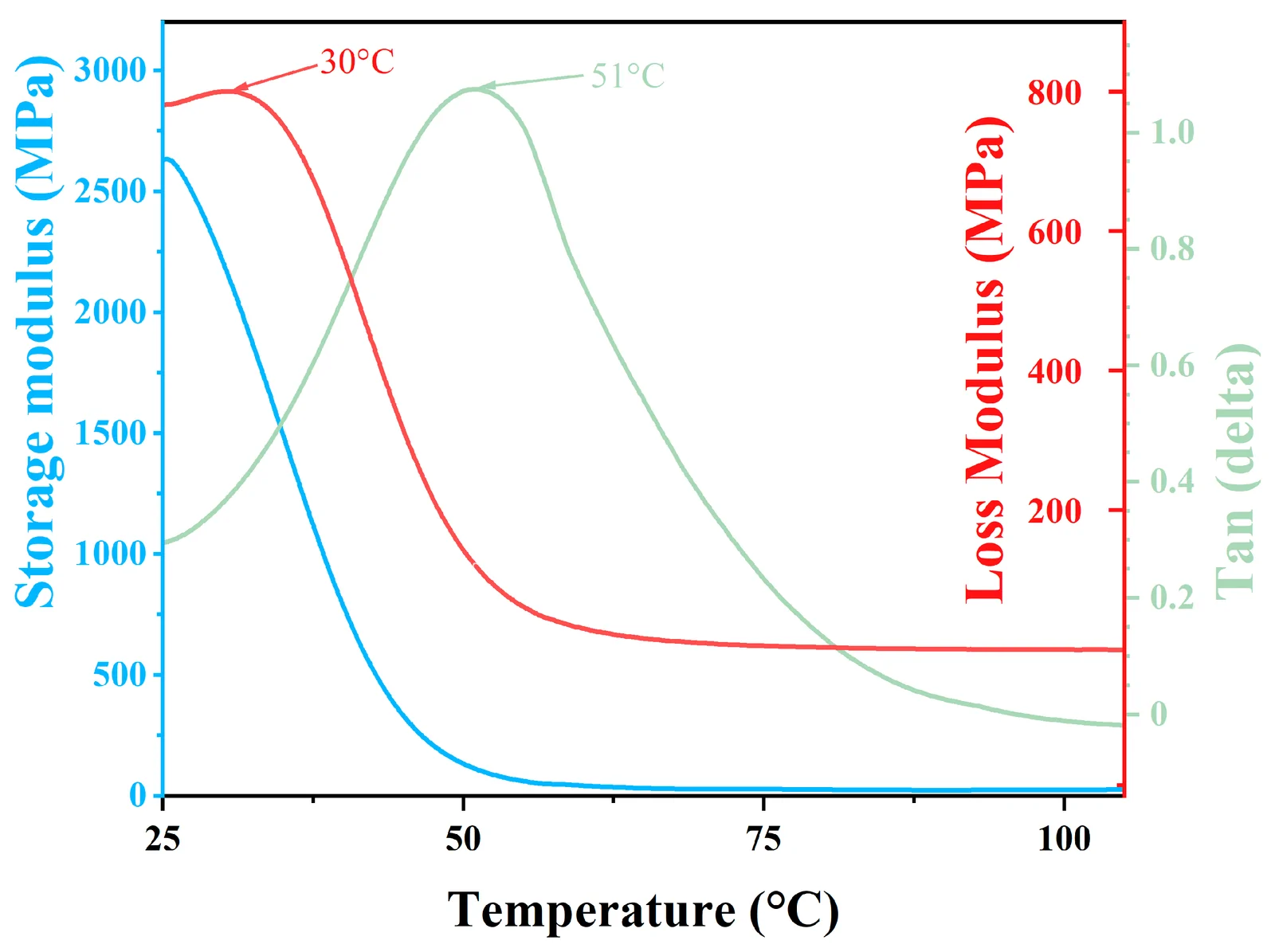
DMA thermograms of Fe_3_O_4_-SMPs–PDCP after the second-step curing.

Dynamic thermomechanical properties are crucial for investigating the behavior of SMP-based ceramic precursors [25,65]. As shown in Figure 9, the storage modulus in the DMA thermograms of the Fe_3_O_4_-SMPs–PDCP composite ceramic precursor exhibits obvious plateau characteristics both at low and high temperatures. At relatively low temperatures, the plateau corresponds to a high storage modulus of 1792 MPa; at relatively high temperatures, the storage modulus decreases significantly to a low positive plateau of 27 MPa. Between these two plateaus, the Fe_3_O_4_-SMPs–PDCP composite ceramic precursor undergoes a shape-memory transition process, resulting in a decrease in the storage modulus by approximately 1.8 orders of magnitude. The sharp decline in storage modulus during the shape memory transition is essential for thermally induced shape memory behavior—only when the modulus is significantly reduced can the material transition from a rigid state to a flexible state, laying the foundation for subsequent shape programming.

To understand these dynamic thermomechanical behaviors from a microscopic structural perspective, it is essential to consider the interfacial interactions among the crosslinked SMP network, the PDCP matrix, and the Fe_3_O_4_ nanoparticles. At macroscopic low temperatures (below T_trans_), the molecular chains within the SMP network are essentially in a “frozen” state. These rigid molecular segments restrict any large-scale macroscopic deformation, resulting in the high storage modulus observed. Furthermore, linking this back to our mechanical tests and MD simulations in Section 3.3, the Fe_3_O_4_ nanoparticles are well accommodated within the polymer network. Their stable non-covalent interfacial interactions with the polymer matrix efficiently transfer stress, which maintains the structural integrity and preserves the high storage modulus of the composite without disrupting its baseline mechanical stability. However, as the temperature increases past T_trans_, the polymer chains transition into a “mobile” state. The SMP molecular chains acquire significant mobility, transforming the material into a highly elastic, soft state. Simultaneously, the elevated temperature weakens the interfacial interactions between the Fe_3_O_4_ nanoparticles and the polymer matrix, dictating the drastic drop in the storage modulus observed in the DMA thermograms.

The shape memory effect of SMPs-based ceramic precursors depends on three key characteristics: the ability to acquire a temporary shape upon heating, the ability to fix the temporary shape upon cooling, and the ability to recover the original preset shape upon reheating. To obtain a new temporary shape, the Fe_3_O_4_-SMPs–PDCP composite ceramic precursor must become sufficiently soft under thermal stimulation to be reshaped under external force. When the temperature exceeds T_trans_ (i.e., 51 °C) and enters the high-temperature region, the material is in a low-modulus, high-elasticity, and soft state with a modulus of 27 MPa, which exactly meets this requirement. Moreover, the modulus remains stable at a low plateau even at a high temperature of 148 °C, ensuring shape-forming capability over a wide temperature range.

To fix the shape, the material must harden upon cooling, which means it must have a high modulus at relatively low temperatures. When the temperature drops below T_trans_, the storage modulus rapidly rebounds to a high level of 1792 MPa, and even reaches a peak value of 2634 MPa at room temperature (25 °C), which can stably fix the temporary shape formed by external force without spontaneous deformation. Upon reheating, the material softens again, and the elastic potential energy stored during the deformation process breaks through the energy barrier, driving the material to recover its original shape. When heated to the transition region near T_trans_, the sharp drop in storage modulus converts the material from a hard state to a soft state, providing space for the release of elastic potential energy. Therefore, the Fe_3_O_4_-SMPs–PDCP composite ceramic precursor must exhibit temperature-dependent modulus changes to possess thermally induced shape memory properties.

The variation law of the storage modulus with temperature indicates that the storage modulus of the Fe_3_O_4_-SMPs–PDCP composite ceramic precursor changes significantly with temperature. At low temperatures, the material has a high storage modulus, indicating that it is in a macroscopically hard state and possesses the ability to fix the temporary shape after cooling. At high temperatures, the storage modulus of the material is relatively low, showing a macroscopically soft state, which meets the conditions for reshaping by external force. This transition of being hard at low temperatures and soft at high temperatures allows the material to be thermally programmed into a new temporary shape, and then recover to its original shape upon thermal stimulation, directly confirming that the Fe_3_O_4_-SMPs–PDCP composite ceramic precursor has excellent thermally induced shape memory properties.

The loss tangent (tanδ) versus temperature curve of the prepared Fe_3_O_4_-SMPs–PDCP composite ceramic precursor exhibits a single-peak symmetric characteristic, and its peak value is usually used to define the T_trans_ of the material. The test results show that the maximum tanδ value of the Fe_3_O_4_-SMPs–PDCP composite ceramic precursor is 1.07, and this peak value accurately corresponds to 51 °C, that is, the T_trans_ of the material is 51 °C.

### 3.7. SMPs–Fe_3_O_4_-PDCP for Self-Deployable Systems

This study develops a thermomagnetically dual-responsive intelligent hinge with broad application prospects in the aerospace field. Aimed at the space constraints of lunar exploration vehicle transportation and storage, the solar panels of the lunar rover adopt a foldable design to reduce volume occupation, thereby meeting the space utilization requirements of deep-space exploration. The thermomagnetically dual-functional response of the hinge can meet the driving demands from different exploration working conditions; among them, the adaptability of the magnetic field characteristics covers energy-constrained scenarios such as the far side of the moon (permanently shadowed region) and the lunar eclipse period.

**Figure 10:**
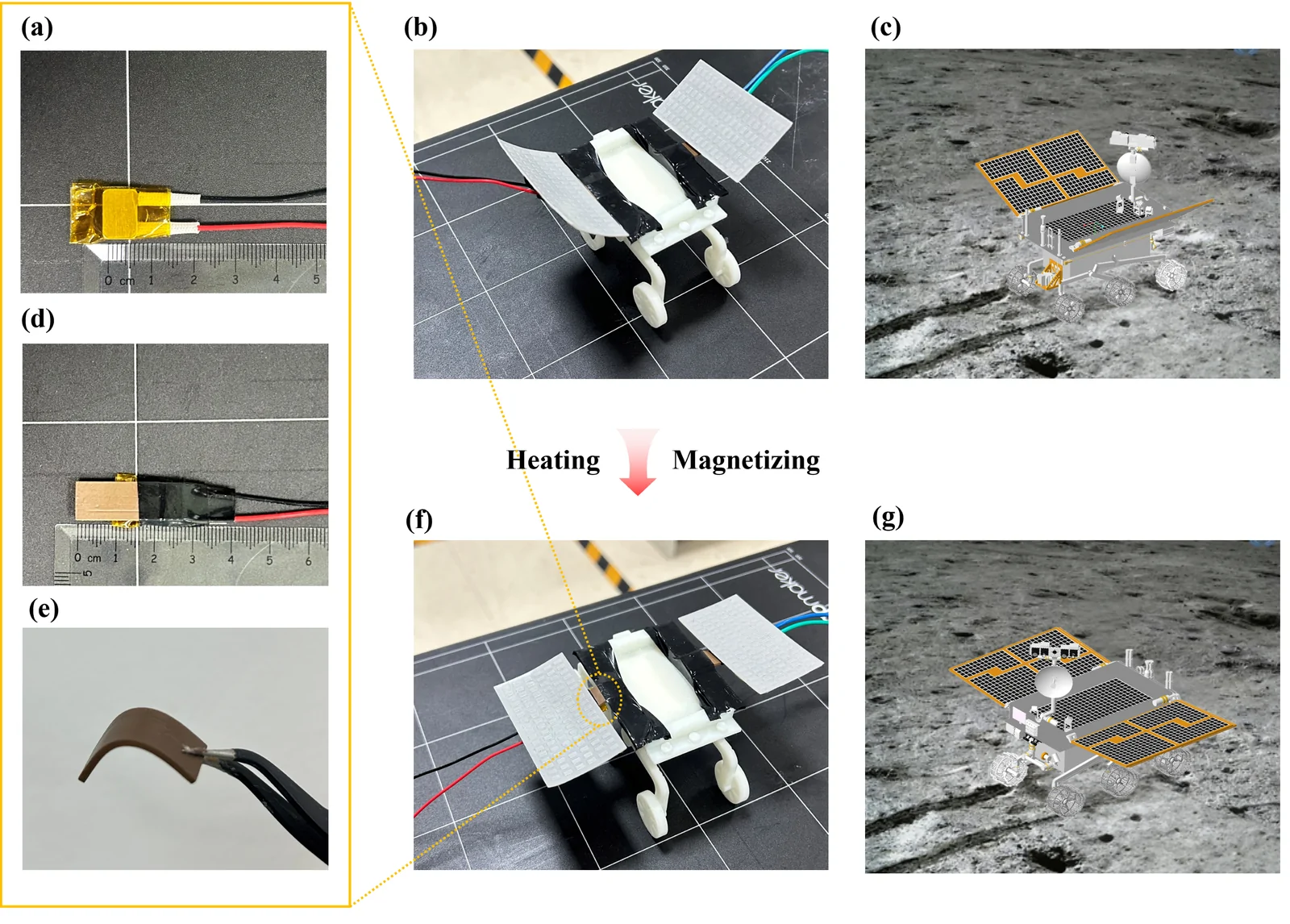
Self-deployable systems fabricated from Fe_3_O_4_-SMPs–PDCP: (**a**,**d**) magnetothermal driving elements of the intelligent hinge; (**e**) bending deformation state of the intelligent hinge; (**b**,**f**) self-deployable system for lunar rovers; (**c**,**g**) schematic diagram of solar panel self-deployment on the lunar surface.

The hinge is programmed to form an initial planar shape and a temporary folded shape (Figure 10). Application of Joule heating or magnetic field stimulation can trigger its shape memory effect, achieving accurate recovery from the temporary shape to the preset deployed configuration. The design of this system verifies the application potential of Fe_3_O_4_-SMPs–PDCP-based dual-stimuli responsive intelligent structures in reconfigurable equipment for deep-space exploration. It expands the boundary of the spatial application of 4D printing technology for this material system and provides a new technical approach for the lightweight and intelligent design of exploration equipment.

## 4. Conclusions

This study has developed a viable SLA-based strategy for high-resolution, programmable, multi-responsive ceramic 4D printing. Through systematic experimental characterization and simulation, the process has been validated and its mechanisms analyzed, effectively overcoming the technical challenges associated with traditional ceramic 4D printing, such as limited stimulus-response modes, restricted manufacturing precision, and poor controllability of deformation. The main conclusions of this study are summarized as follows:

(1) A ceramic precursor slurry composed of SMPs and Fe_3_O_4_ was successfully developed for printing using high-precision photopolymerization processes. The established photothermal stepwise crosslinking and curing process enables high-resolution forming accuracy and programmable deformation of the precursor components. By leveraging the heat-generating properties of Fe_3_O_4_ nanoparticles under alternating magnetic fields and the shape memory effect of thermosetting SMPs, this composite system enables the programmable dynamic deformation of ceramic precursors when stimulated by magnetic or thermal fields, offering a feasible pathway for contactless intelligent actuation of ceramic materials.
(2) By combining macroscopic performance testing with microscopic mechanism analysis, we systematically analyzed the mechanical properties and multi-response behavior of the composite precursors. Molecular dynamics simulation revealed the interfacial bonding patterns between the polymer and inorganic particles at the microscopic scale, providing important theoretical support for the functional design and performance optimization of composite ceramic precursors; the simulation results were consistent with the conclusions of the mechanical experiments. Concurrently, multi-dimensional test results confirm that this composite system possesses excellent suitability and application potential in the field of multifunctional ceramic 4D printing.
(3) As a proof of concept, the application potential of this material system in self-deployable structures was demonstrated, offering a novel approach to the high-precision, multifunctional manufacturing of smart ceramic components, with broad application prospects in fields such as aerospace deployable mechanisms and non-contact biomedical devices.

Overall, this study established a comprehensive technical framework for ceramic precursor 4D printing that integrates slurry formulation optimization, printing process parameter control, high-precision shaping via photothermal stepwise curing, and programmable multi-response deformation. This strategy effectively broadens the multifunctional fabrication pathways for smart ceramic components and provides a reliable technical foundation for the design and development of self-deployable intelligent structures.

## Data Availability

The original contributions presented in this study are included in the article. Further inquiries can be directed to the corresponding author.
